# Personalized Diabetes Treatment Support Using Large Language Models Fine-Tuned on Electronic Health Records: Development and Evaluation Study

**DOI:** 10.2196/71541

**Published:** 2026-02-09

**Authors:** Shengyang He, Yu Zhang, Jiaxi Li

**Affiliations:** 1 Department of Clinical Laboratory Medicine, Jinniu Maternity and Child Health Hospital of Chengdu, Chengdu, 610041, China, 86 18227387870; 2 Information Center, West China Hospital of Sichuan University, Chengdu, Sichuan, China

**Keywords:** GLM4-9B, artificial intelligence, AI, large language model, diabetes, electronic health record, EHR

## Abstract

**Background:**

Effective diabetes management requires individualized treatment strategies tailored to patients’ clinical characteristics. With recent advances in artificial intelligence, large language models (LLMs) offer new opportunities to enhance clinical decision support, particularly in generating personalized recommendations.

**Objective:**

This study aimed to develop and evaluate an LLM-based outpatient treatment support system for diabetes and examine its potential value in routine clinical decision-making.

**Methods:**

Three compact LLMs (Llama 3.1-8B, Qwen3-8B, and GLM4-9B) were fine-tuned on deidentified outpatient electronic health records using a parameter-efficient low-rank adaptation approach. The optimized models were embedded into a prototype hospital information system via a retrieval-augmented generation framework to generate individualized treatment recommendations, laboratory test suggestions, and medication prompts based on demographic and clinical data.

**Results:**

Among the models evaluated, the fine-tuned GLM4-9B demonstrated the strongest performance, producing clinically reasonable treatment plans and appropriate laboratory test recommendations and medication suggestions. It achieved a mean Bilingual Evaluation Understudy for 4-grams score of 67.93 (SD 2.74) and mean scores of 44.30 (SD 3.91) for Recall-Oriented Understudy for Gisting Evaluation for overlap of unigrams, 27.34 (SD 1.85) for Recall-Oriented Understudy for Gisting Evaluation for overlap of bigrams, and 37.67 (SD 2.88) for Recall-Oriented Understudy for Gisting Evaluation for Longest Common Subsequence.

**Conclusions:**

The fine-tuned GLM4-9B shows strong potential as a clinical decision support tool for personalized diabetes care. It can provide reference recommendations that may improve clinician efficiency and support decision quality. Future work should focus on enhancing medication guidance, expanding data sources, and improving adaptability in cases involving complex comorbidities.

## Introduction

Diabetes is a chronic metabolic disorder characterized by elevated blood glucose levels, which over time can cause serious damage to the heart, blood vessels, eyes, kidneys, and nervous system. The most common is type 2 diabetes, which accounts for approximately 90% to 95% of diabetes cases [1] and affects mainly adults. According to the World Health Organization, approximately 422 million people worldwide have diabetes, most in low- and middle-income countries, and 1.5 million people die each year as a direct result of diabetes [2]. The incidence and prevalence of diabetes have been steadily increasing over the last few decades [1]. As patients with diabetes require long-term medication to control blood glucose levels and prevent complications [3], they can face several challenges during the treatment process, such as medication selection, dosage adjustment, and management of adverse effects. Failure to address these issues in a timely manner can compromise the efficacy of medication and even pose a threat to patients’ lives [4]. Therefore, people with diabetes need timely medication advice, health education, and nutrition support to help them use their medicines correctly, safely, and effectively, thereby improving adherence and quality of life. To better serve patients and increase the efficiency of health care professionals, we aim to optimize the management of patients with diabetes through the application of artificial intelligence.

With the significant success of ChatGPT in tasks related to understanding and generating humanlike responses [5], large language models (LLMs) have attracted considerable attention. They have shown strong performance in various natural language processing tasks and the ability to generalize to unfamiliar tasks, demonstrating their potential as a unified solution for natural language understanding, text generation, and dialogue. Although ChatGPT has shown promising results in medical document summarization and decision support [6,7], as well as in passing the US Medical Licensing Examination Steps 1 and 2 [8], the exploration of these broad-domain LLMs in the medical field is still relatively limited [9]. Currently, there is a lack of specifically trained LLMs in the field of health care. To address this gap, we plan to fine-tune an LLM using deidentified data from patients with diabetes with the aim of exploring its application in diabetes management. In addition, harnessing the potential of LLMs will open up new opportunities for medical research and practice and drive advances and innovation in health care technology.

## Methods

### Ethical Considerations

This retrospective study used encrypted and deidentified data from West China Hospital involving no patient privacy–sensitive information or clinical interventions. Data extraction and study procedures were approved by the ethics committee of West China Hospital, Sichuan University (approval 2024-126), in accordance with informed consent requirements.

### Data Collection

This study used the big data integration platform of West China Hospital as the primary source of data [10]. We collected electronic health record data from patients diagnosed with diabetes who visited the outpatient department from January 2022 to February 2022. The collected data included information such as the patients’ department of visit, age, gender, chief concern, present illness history, and diagnosis, which served as input for the model. In addition, we obtained data on patients’ outpatient medications, laboratory test items, examinations, and physician opinions, which were used as model outputs. Patient data with missing chief concerns and present illness history were excluded to ensure data quality and usability. Furthermore, we collected an additional set of data from 300 patients (visiting in March 2022) as a test set to evaluate the performance of the optimized model.

### Data Preprocessing

Before model training, we performed systematic preprocessing on the collected patient data, including data cleaning, standardization, and anonymization. Data cleaning involved removing missing values, outliers, and duplicate records to ensure the integrity and reliability of the model inputs. Standardization ensured consistency in format, units, and value ranges across different data types. For text data, including chief concerns, present illness history, examinations, and laboratory test results, we applied cleaning, terminology unification, and segmenting of key information while removing sensitive information to protect patient privacy. The processed data were then organized into a question-answer format (instruction-input-output) suitable for LLM fine-tuning: patient basic information and clinical text (eg, department, gender, age, diagnosis, chief concern, and present illness history) were used as the input, and corresponding medical orders, examinations, recommended laboratory tests, and treatment recommendations were integrated as the output (shown in Textbox 1). This approach ensured that both structured and unstructured information was effectively used for model training. The dataset was then split into training and test sets in an 8:2 ratio.

Textbox 1. **Data example for large language model fine-tuning in clinical diabetes recommendations.**

**Instruction**

- “You are a diabetes clinical recommendation model. You will receive the patient’s basic information and laboratory results, and you are expected to provide tailored clinical recommendations for the patient.”

**Input**

- Department: endocrinology ward
- Sex: male
- Age: 55 years
- Diagnosis: type 2 diabetes mellitus with hypertension
- Chief concern: polydipsia; polyuria for 6 months
- Present illness history: “The patient reports excessive thirst and frequent urination over the past six months, with a weight loss of approximately 3 kg. History of hypertension for 5 years, blood pressure poorly controlled. Recent fasting blood glucose ranges from 8–10 mmol/L, postprandial blood glucose up to 12 mmol/L. The patient is not on regular insulin or oral hypoglycemic therapy.”

**Output**

- Provision of a blood glucose monitoring device and test strips
- Laboratory tests: fasting plasma glucose, 2-hour postprandial glucose, glycated hemoglobin, liver and renal function, and lipid panel
- Examinations: fundoscopy, foot examination, and electrocardiogram
- Treatment recommendations: adjust insulin or oral hypoglycemic agents according to blood glucose levels, monitor blood pressure, and implement dietary and exercise interventions

### Model Selection

In this study, we compared 3 LLMs for clinical text prediction tasks: Llama 3.1-8B (Meta AI) [11], Qwen3-8B (Alibaba Cloud) [12], and GLM4-9B (THUDM/Z.ai) [13]. Llama 3.1-8B is an open-source model optimized for efficient generalization and complex text understanding. Qwen3-8B is a multilingual LLM with strong capabilities in both structured and unstructured medical text processing. GLM4-9B, developed based on the general language model architecture, is designed for bilingual question-answering tasks and supports local deployment through model quantization. All models were fine-tuned and evaluated using a single H100 graphics processing unit (NVIDIA). Smaller model sizes were chosen to facilitate clinical deployment and wider adoption in practical settings while still maintaining competitive performance for downstream tasks.

### Model Fine-Tuning

To adapt the selected LLMs for clinical treatment recommendation tasks, we used a parameter-efficient fine-tuning strategy that combines instruction-based prompting with low-rank adaptation (LoRA) [14]. Instruction templates were designed to explicitly guide the model in interpreting patient information and generating clinically appropriate treatment suggestions. Building on this, LoRA was applied to the attention layers to enable efficient task-specific adaptation while updating only a small number of parameters. During fine-tuning, we explored multiple hyperparameter configurations, including learning rate, batch size, and LoRA-specific parameters such as rank and scaling factor. Model performance was assessed on a held-out validation set using both automatic text generation metrics (eg, Bilingual Evaluation Understudy [15] and Recall-Oriented Understudy for Gisting Evaluation [16]) and clinically oriented evaluation criteria, including the correctness and appropriateness of the recommended treatments. This combined approach provided a balanced trade-off between computational efficiency and clinical relevance, offering practical guidance for deploying LLM-based treatment recommendation systems in real-world clinical settings.

### Physician Assessment of Recommendations

To ensure a rigorous and clinically meaningful evaluation of our LLM-generated diabetes treatment recommendations, we implemented a structured, multidimensional physician assessment protocol. Six dimensions were assessed: treatment appropriateness, medication accuracy, relevance of suggested examinations, safety, logical reasoning, and overall clinical usefulness. Each dimension was rated using a standardized 5-point Likert scale [17] ranging from 1 (“completely unreasonable or not useful”) to 5 (“fully reasonable and clinically valuable”). Five board-certified endocrinologists (each with more than 5 years of independent clinical practice) served as expert raters. All assessments were performed independently and in a fully blinded manner: raters were unaware of which LLM produced each recommendation and were prohibited from discussing cases. Each physician reviewed the same 300 treatment recommendations generated by the 3 LLMs, including both base and fine-tuned versions. Recommendations included medication plans, dosage adjustments, and suggested laboratory tests. This protocol ensured a systematic, reproducible, and clinician-centered evaluation of model outputs, enabling identification of potential risks and areas requiring refinement.

### Retrieval-Augmented Generation and Agent-Assisted Clinical Data Processing

**Figure 1.**
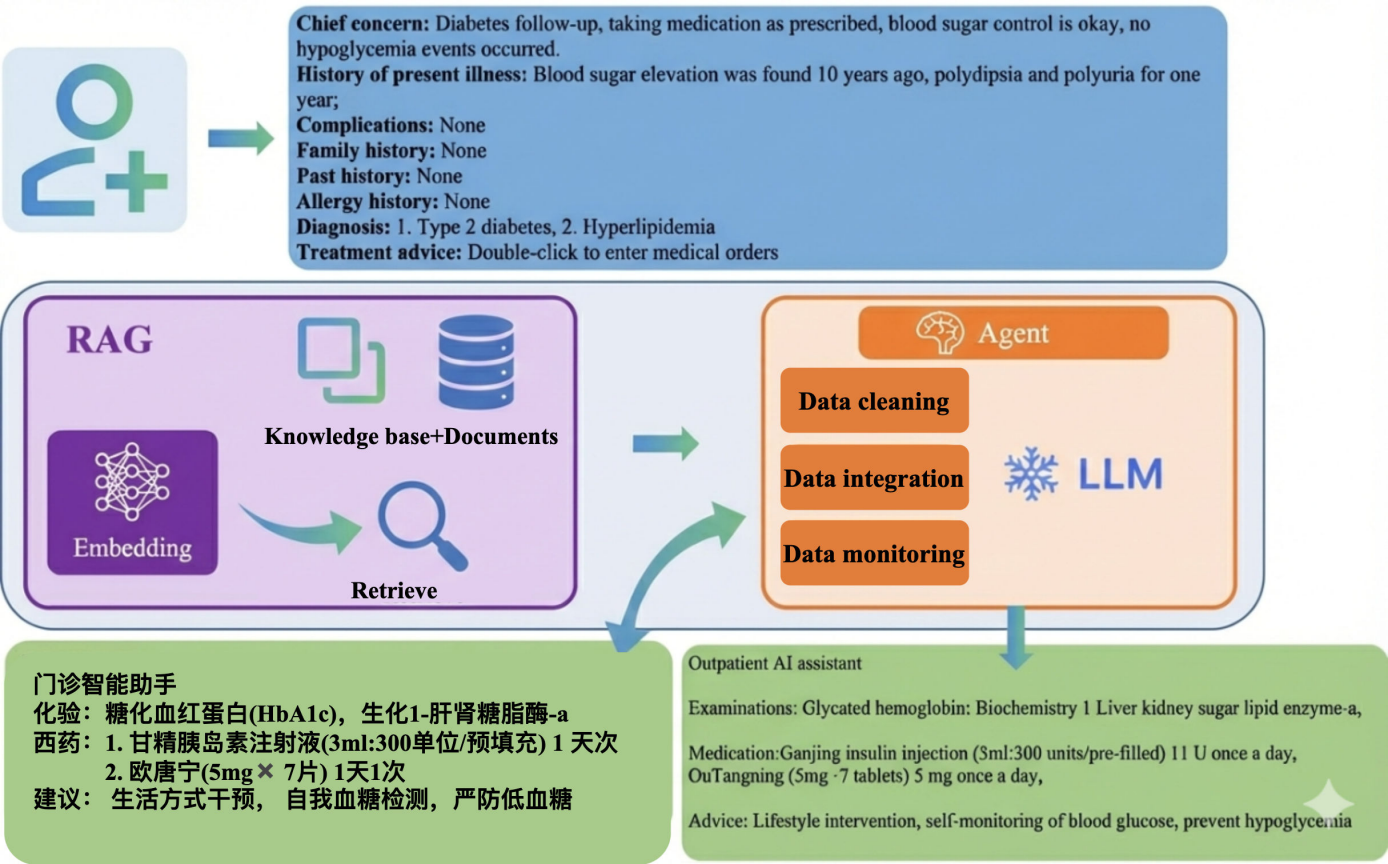
Schematic workflow of the clinical application system integrating retrieval-augmented generation (RAG)– and agent-driven data management. AI: artificial intelligence; LLM: large language model.

We implemented a retrieval-augmented generation (RAG) [18] framework to integrate hospital knowledge resources, including the medical order database, diabetes treatment guidelines, and clinical protocols, enabling the model to dynamically retrieve relevant knowledge during treatment recommendations and provide context-specific, up-to-date clinical information. An agent system was developed to interface with the hospital information system. It handles data cleaning, integration, and monitoring by extracting and standardizing patient data, consolidating heterogeneous sources into a structured format suitable for RAG, and ensuring data integrity and consistency. By combining RAG with agent-driven data management, the system efficiently leverages internal knowledge bases to support clinically grounded, accurate, and interpretable treatment recommendations. The overall workflow of this clinical application system is illustrated in Figure 1.

## Results

### Overview

**Figure 2.**
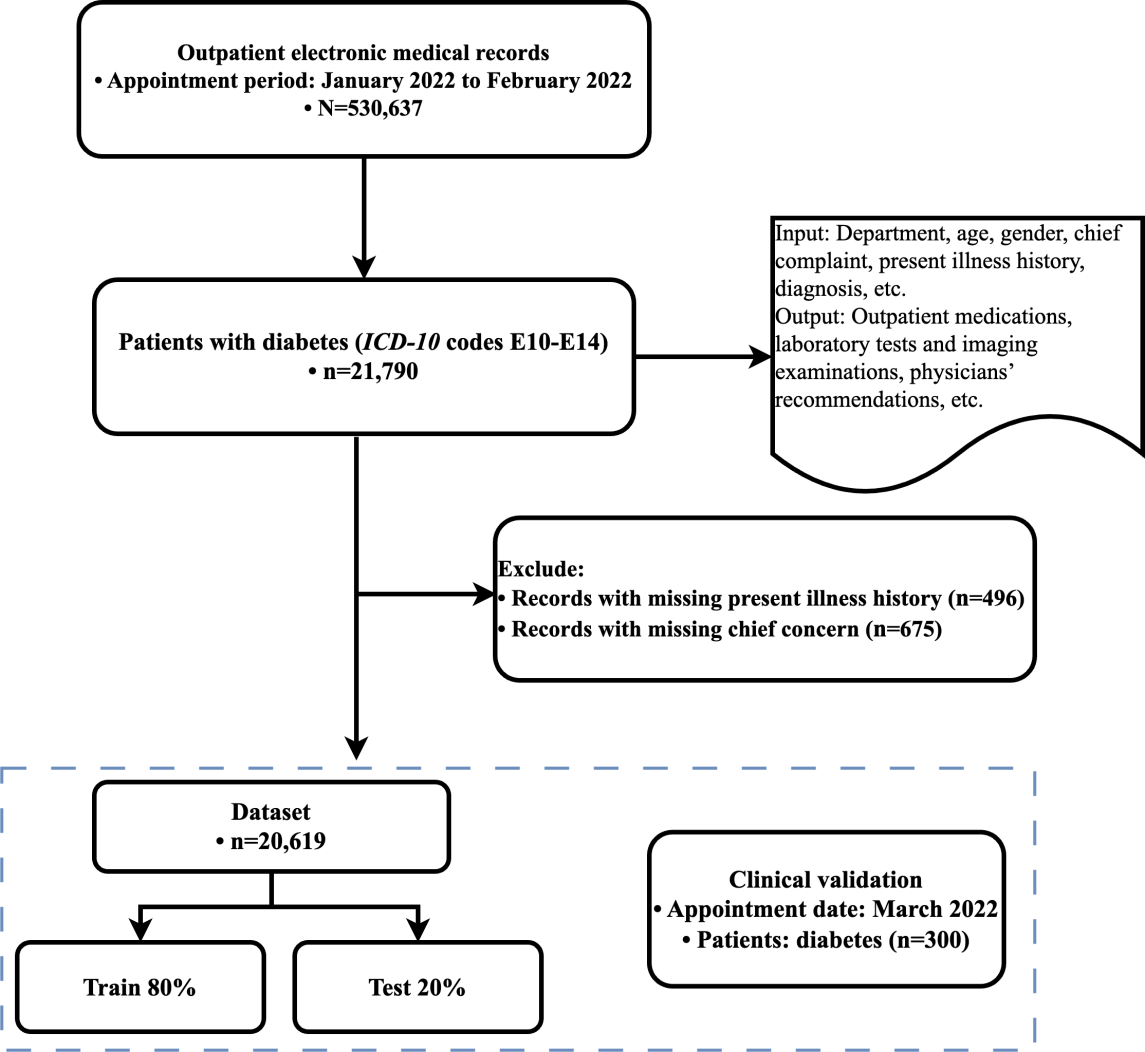
Flowchart of patient inclusion and exclusion criteria

As shown in Figure 2, the final dataset comprised 20,619 patients, with 80% allocated for model training and 20% held out as a test set for performance evaluation. Figure 2 illustrates the data collection process.

**Figure 3.**
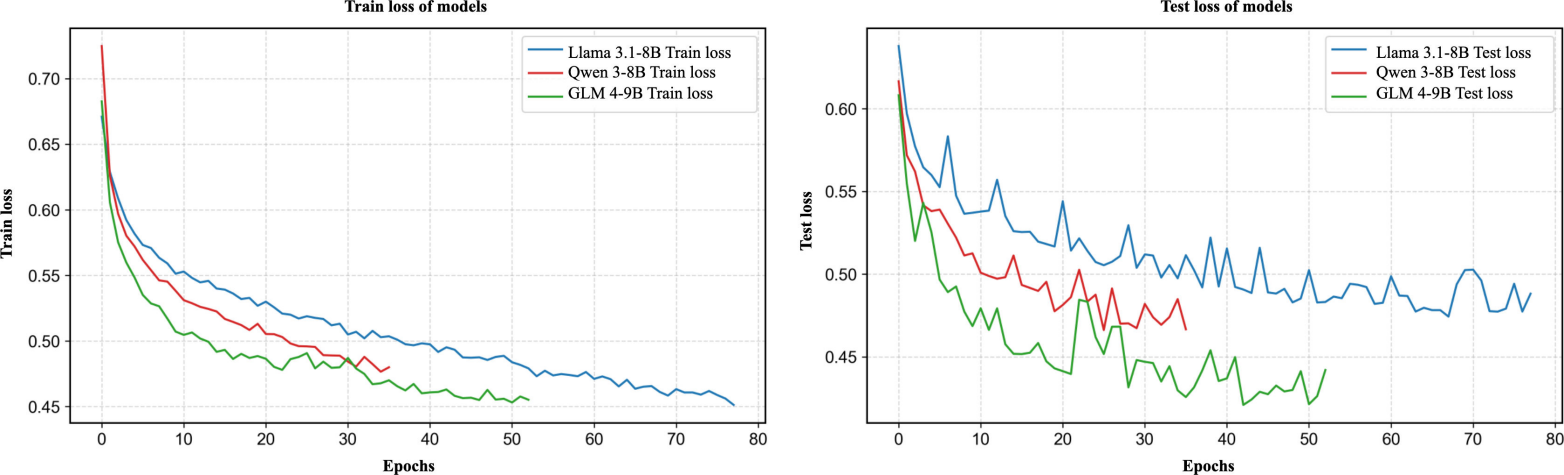
Training and test loss curves for Llama 3.1-8B, Qwen3-8B, and GLM4-9B.

All 3 LLMs—Llama 3.1-8B, Qwen3-8B, and GLM4-9B—were evaluated before and after LoRA fine-tuning. Their training and test loss curves are shown in Figure 3, and model performance was assessed using both automatic text generation metrics and clinical physician evaluations.

Table 1 summarizes the comparative performance of the baseline and fine-tuned models measured using Bilingual Evaluation Understudy for 4-grams (BLEU-4), Recall-Oriented Understudy for Gisting Evaluation for overlap of unigrams (ROUGE-1), Recall-Oriented Understudy for Gisting Evaluation for overlap of bigrams (ROUGE-2), and Recall-Oriented Understudy for Gisting Evaluation–Longest Common Subsequence (ROUGE-L). Before fine-tuning, the 3 base models demonstrated moderate capability in generating clinically relevant recommendations, with mean BLEU-4 scores ranging from 45.13 (SD 1.98) to 50.84 (SD 1.87) and ROUGE-L scores ranging from 9.83  (SD 0.54) to 13.90 (SD 0.74). Among the base models, GLM4-9B achieved the highest performance across all metrics. After fine-tuning, all models showed significant gains in both lexical similarity and content relevance. The BLEU-4 mean score increased by 14.48 points for Llama 3.1-8B, 15.95 points for Qwen3-8B, and 17.09 points for GLM4-9B. ROUGE-1 and ROUGE-L exhibited similar patterns, with improvements exceeding 20 points for all 3 models. The fine-tuned GLM4-9B outperformed all models, achieving the highest BLEU-4 (mean 67.93, SD 2.74), ROUGE-1 (mean 44.30, SD 3.91), ROUGE-2 (mean 27.34, SD 1.85), and ROUGE-L (mean 37.67, SD 2.88) scores.

**Table 1.** Performance analysis based on Bilingual Evaluation Understudy for 4-grams (BLEU-4) and Recall-Oriented Understudy for Gisting Evaluation scores.

Model	BLEU-4, mean (SD)	ROUGE-1^a^, mean (SD)	ROUGE-2^b^, mean (SD)	ROUGE-L^c^, mean (SD)
Base Llama 3.1-8B	45.13 (1.98)	12.55 (0.68)	3.12 (0.21)	9.83 (0.54)
Base Qwen3-8B	47.92 (2.11)	15.33 (0.75)	4.46 (0.26)	11.77 (0.63)
Base GLM4-9B	50.84 (1.87)	17.92 (0.82)	5.71 (0.31)	13.90 (0.74)
Fine-tuned Llama 3.1-8B	59.61 (3.21)	36.12 (3.44)	22.48 (1.78)	31.44 (2.93)
Fine-tuned Qwen3-8B	63.87 (3.18)	40.58 (3.72)	25.01 (1.92)	34.80 (3.12)
Fine-tuned GLM4-9B	67.93 (2.74)	44.30 (3.91)	27.34 (1.85)	37.67 (2.88)

a ROUGE-1: Recall-Oriented Understudy for Gisting Evaluation for overlap of unigrams.

b ROUGE-2: Recall-Oriented Understudy for Gisting Evaluation for overlap of bigrams.

c ROUGE-L: Recall-Oriented Understudy for Gisting Evaluation–Longest Common Subsequence.

Regarding the clinical evaluation, the performance of the original models and their LoRA fine-tuned versions is summarized in Textbox 2.

Textbox 2. **Performance of the original models and their low-rank adaptation fine-tuned versions.**

**Input**

- Department: endocrinology and metabolism medical unit
- Gender: male
- Age: 68 years
- Diagnosis: type 2 diabetes; hyperlipidemia
- Chief concern: diabetes follow-up visit
- Medical history: diabetes for 2 or more years

**Real clinical data output**

- Laboratory tests: liver and kidney function, glucose and fat-a, and glycated hemoglobin (HbA_1c_; biochemistry 2)
- Medication: 11 units once a day of Ganjing insulin injection (3 mL: 300 prefilled units), 5 mg of OuTangning once a day (5 mg × 7 tablets), and 10 mg of atorvastatin calcium tablets once every night (10 mg × 28 tablets)

**Base GLM4-9B output**

- “A patient with diabetes attending a follow-up visit requires a comprehensive medical history review and physical examination to better assess their condition and guide treatment planning. According to your description, the patient has type 2 diabetes and hyperlipidemia. Type 2 diabetes is a chronic condition that necessitates long-term management, while hyperlipidemia is a common metabolic disorder that can negatively impact cardiovascular health and increase the risk of cardiovascular disease. During follow-up, the patient should monitor blood glucose levels and adjust management strategies—including medication, diet, and exercise—accordingly. Regular monitoring of blood lipid levels is also necessary, with treatment plans tailored based on the results. Common treatment strategies for diabetes include oral medications, insulin therapy, blood glucose monitoring, and dietary control. Successful management requires active patient engagement, adherence to lifestyle recommendations, and consistent follow-up to maintain stable and healthy blood glucose levels.”

**Fine-tuned GLM4-9B output**

- Laboratory tests: HbA_1c_ and liver and kidney function tests, lipase A, fasting insulin, 2-hour postprandial insulin, fasting plasma glucose, 2-hour postprandial C-peptide, and 2-hour postprandial plasma glucose (biochemistry 1)
- Medication: 11 units once daily of Ganjing insulin injection (3 mL: 300 units/prefilled) and 5 mg of OuTangning once daily (5 mg × 7 tablets)
- Physician’s recommendation: regular follow-up visits are advised to monitor treatment response and adjust therapy as needed

### Clinical Evaluation

Table 2 shows the results of 5 endocrinologists’ evaluation of the 300 treatment recommendations generated by the 3 fine-tuned LLMs. Overall, the mean scores indicated that most recommendations were clinically relevant to diabetes management (mean usefulness scores above 4 for all fine-tuned models), whereas the base models received substantially lower ratings. Approximately 10% of the recommendations (32/300, 10.7%) were judged by at least one rater as potentially risky (eg, inappropriate escalation or overtreatment), underscoring the need for human oversight.

**Table 2.** Physician assessment of recommendations.

Model	Treatment appropriateness (1-5), mean (SD)	Medication accuracy (1-5), mean (SD)	Relevance (1-5), mean (SD)	Safety (1-5), mean (SD)	Logical reasoning (1-5), mean (SD)	Usefulness (1-5), mean (SD)
Base GLM4-9B	3.28 (0.38)	2.96 (0.37)	3.52 (0.34)	3.60 (0.53)	3.60 (0.50)	3.44 (0.30)
Fine-tuned GLM4-9B	4.72 (0.31)	4.48 (0.43)	4.76 (0.36)	4.66 (0.47)	4.78 (0.20)	4.78 (0.16)
Base Llama 3.1-8B	2.50 (0.53)	2.80 (0.70)	2.94 (0.18)	2.86 (0.29)	2.54 (0.36)	2.60 (0.54)
Fine-tuned Llama 3.1-8B	4.58 (0.47)	4.68 (0.43)	4.70 (0.42)	4.40 (0.49)	4.36 (0.57)	4.60 (0.39)
Base Qwen3-8B	3.42 (0.42)	2.98 (0.41)	3.00 (0.85)	3.14 (0.62)	3.32 (0.48)	3.24 (0.51)
Fine-tuned Qwen3-8B	4.50 (0.71)	4.26 (0.50)	4.16 (0.78)	3.80 (0.37)	4.44 (0.55)	4.44 (0.38)

**Figure 4.**
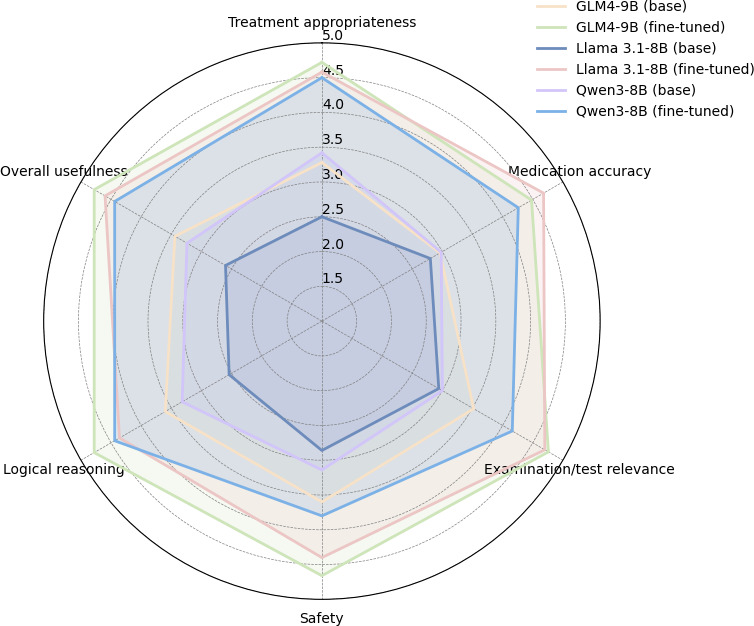
Radar chart illustrating the multidimensional physician assessment scores of treatment recommendations generated by Llama 3.1-8B, Qwen3-8B, and GLM4-9B before and after low-rank adaptation fine-tuning.

As shown in Figure 4, the radar chart highlights the multidimensional improvements achieved through LoRA fine-tuning across all 6 evaluation metrics. For all 3 models, the fine-tuned versions consistently exhibited higher scores than their base counterparts. Among the models, GLM4-9B (fine-tuned) achieved the highest performance across nearly all dimensions, particularly in treatment appropriateness, relevance, and overall usefulness.

## Discussion

### Principal Findings

This study systematically evaluated the performance of 3 LLMs (Llama 3.1-8B, Qwen3-8B, and GLM4-9B) on a clinical treatment recommendation task for diabetes and explored a parameter-efficient optimization strategy combining instruction-based prompting and LoRA fine-tuning. It should be noted that all 3 models are general-purpose open-source LLMs and were not specifically fine-tuned on medical data, serving as preliminary baselines for clinical recommendation tasks. The untuned base models achieved moderate performance in generating clinically relevant recommendations, with mean BLEU-4 scores ranging from 45.13 to 50.84 and mean ROUGE-L scores ranging from 9.83 to 13.90, among which the base GLM4-9B model performed best across metrics. These findings indicate that even relatively small models possess nontrivial text generation capabilities but exhibit limitations in content relevance and accuracy for specialized clinical tasks. After LoRA fine-tuning, all models showed substantial improvements on both automatic evaluation metrics and clinician assessments. BLEU-4 scores increased by approximately 14 to 17 absolute points on average, whereas ROUGE-1 and ROUGE-L scores improved by more than 20 points, suggesting that fine-tuning effectively enhanced lexical similarity and information completeness in the generated texts. In clinician evaluations, scores increased markedly across the 6 dimensions—therapeutic appropriateness, medication accuracy, examination relevance, safety, logical reasoning, and overall usefulness. Among them, fine-tuned GLM4-9B achieved the best performance on key dimensions such as therapeutic appropriateness, relevance, and overall usefulness, whereas fine-tuned Llama 3.1-8B and Qwen3-8B also demonstrated varying degrees of improvement (Figure 4). The radar chart clearly illustrates these multidimensional gains, underscoring the effectiveness of combining LoRA with instruction-based prompting to enhance clinical applicability. Furthermore, by leveraging an RAG framework and an agent-based data processing and knowledge retrieval pipeline (Figure 1), the models can dynamically access up-to-date institutional clinical guidelines, medication order databases, and diabetes treatment standards, thereby enabling individualized, context-aware clinical recommendations. This approach not only improves the accuracy and safety of the generated suggestions but also strengthens their interpretability and practical usability in clinical settings. After repeated debugging and testing of the model, we found that, for a subset of patients with complex medical records, the model output can be potentially harmful and fail to assist health care professionals in their treatment. This situation can occur for the following reasons:

- Data imbalance or sample bias [19]: the model may have been trained on an overrepresentation of certain types of patient records, leading to an inadequate understanding of other types of patients. This bias can lead to inaccurate or harmful treatment recommendations for certain patients.
- Unknown or rare scenarios [20]: if the model encounters unfamiliar or infrequent situations during training, it may struggle to make accurate predictions or appropriate recommendations. Complex patient records often contain such unknown scenarios, rendering the model’s output ineffective.
- Limitations of the model [21]: the model may have inherent design or training limitations that prevent it from adequately accounting for factors specific to complex medical records. As a result, the model’s outputs may lack accuracy or reliability in these cases. Additionally, the fact that the models are not medically fine-tuned may contribute to these limitations, highlighting the importance of exploring medically fine-tuned LLMs and larger, multi-institutional datasets in future research.
- The size of the training data has a direct impact on the performance of large models. Therefore, it is crucial for us to explore methods to increase the scale of our dataset (Multimedia Appendix 1).

### Limitations

This study has several limitations. First, the proposed framework has been evaluated only in a controlled experimental setting and has not yet been prospectively validated in real-world clinical workflows. As such, its clinical effectiveness and operational impact remain to be systematically assessed. Second, we only used relatively small-scale models, which have substantially fewer parameters than larger frontier models such as DeepSeek R1 [22], Qwen3-32B, or Llama 3-65B. While the smaller models reduce computational requirements and facilitate low-cost intranet deployment, they may limit performance and generalization compared with larger models. Third, the training data for this study were collected from a single medical institution and are relatively limited in size, which may affect the robustness and external validity of the results. It is important to understand that, while LLMs often perform well in many scenarios, they may have limitations when dealing with complex medical records. Therefore, model outputs should not be the sole basis for decision-making. Health care professionals should rely on their expertise and clinical judgment and integrate model outputs with comprehensive assessments to make informed decisions [23]. Additionally, we plan to fine-tune domain-specific medical LLMs (such as MedGemma 27B [24]) and explore the integration of medical knowledge graphs to incorporate structured clinical knowledge, enhance model reasoning [25], and further improve the consistency and reliability of the generated recommendations. Future research will also focus on leveraging larger models and multi-institutional datasets to strengthen model performance, generalizability, and robustness.

### Conclusions

Overall, this study validates the feasibility and effectiveness of combining small-scale LLMs with LoRA fine-tuning and an RAG- and agent-assisted data processing and knowledge retrieval strategy for clinical treatment recommendations in diabetes. The fine-tuned models not only achieved superior performance on automated text generation metrics but also generated treatment recommendations deemed safe, clinically appropriate, and of substantial reference value in structured clinician evaluations. These findings provide a viable pathway for the responsible deployment of LLMs in real-world medical applications. Future research should scale up training datasets; extend validation to a broader range of disease entities; and incorporate longitudinal real-world evidence to further assess long-term clinical effectiveness, safety, and generalizability.

## Supplementary material

10.2196/71541 Multimedia Appendix 1 Supplementary material demonstrating the outputs of the large language models before and after fine-tuning. Screenshots illustrate how different fine-tuning strategies (P-tuning and low-rank adaptation) improve clinical response accuracy; provide structured treatment suggestions; and reduce harmful or inaccurate recommendations in typical, complex, and rare patient scenarios.

## Abbreviations

BLEU-4: Bilingual Evaluation Understudy for 4-grams
LLM: large language model
LoRA: low-rank adaptation
RAG: retrieval-augmented generation
ROUGE-1: Recall-Oriented Understudy for Gisting Evaluation for overlap of unigrams
ROUGE-2: Recall-Oriented Understudy for Gisting Evaluation for overlap of bigrams
ROUGE-L: Recall-Oriented Understudy for Gisting Evaluation–Longest Common Subsequence
