# Correction: Personalized Diabetes Treatment Support Using Large Language Models Fine-Tuned on Electronic Health Records: Development and Evaluation Study

**DOI:** 10.2196/93482

**Published:** 2026-03-26

**Authors:** Shengyang He, Yu Zhang, Jiaxi Li

**Affiliations:** 1 Department of Clinical Laboratory Medicine, Jinniu Maternity and Child Health Hospital of Chengdu, Chengdu, 610041, China, 86 18227387870; 2 Information Center, West China Hospital of Sichuan University, Chengdu, Sichuan, China

In “Personalized Diabetes Treatment Support Using Large Language Models Fine-Tuned on Electronic Health Records: Development and Evaluation Study” [1], the authors noted one error.

In the author list, the first author’s name was changed from the following:

ShenYang He

The name now reads:

Shengyang He

The correction will appear in the online version of the paper on the JMIR Publications website, together with the publication of this correction notice. Because this was made after submission to PubMed, PubMed Central, and other full-text repositories, the corrected article has also been resubmitted to those repositories.
