# Supplementary material for: Personalized Diabetes Treatment Support Using Large Language Models Fine-Tuned on Electronic Health Records: Development and Evaluation Study
Source: JMIR Form Res. 2026 Feb 9;10:e71541. doi: 10.2196/71541 (PMC12885450; doi:10.2196/71541)
Supplement: Multimedia Appendix 1 [file formative-v10-e71541-s001.docx]

Supplementary material

1. The display of results after fine-tuning the model, with screenshots showing the actual effects in the images.

| Input:  ##Department: Endocrinology and Metabolism Medical Unit  ##Gender: Male  ##Age: 68 years old  ##Diagnosis: Type 2 diabetes; hyperlipidemia  ##Chief complaint：Diabetes follow-up visit  ##Medical history：Diabetes for 2 more years |
| --- |

Origin ChatGLM results:


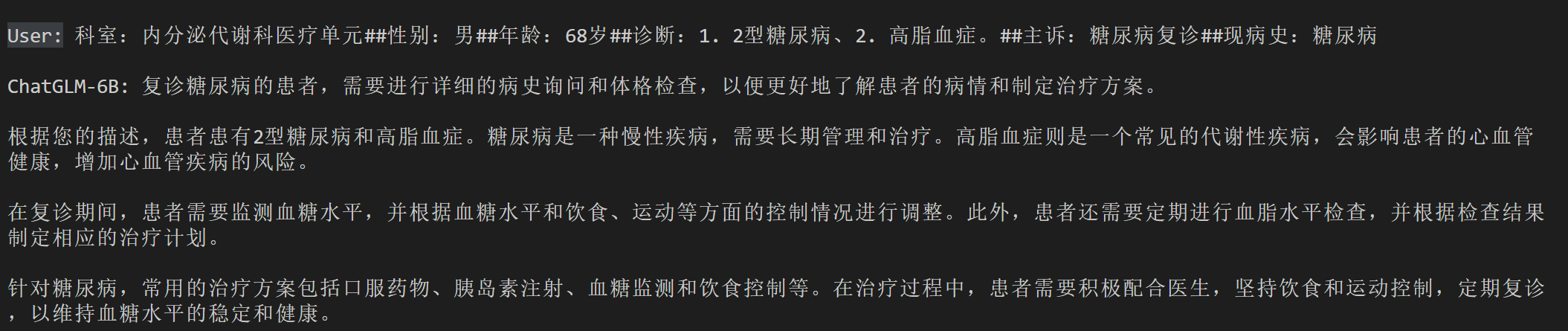


| **Output:**  A patient with diabetes who comes for a follow-up visit needs to undergo a detailed medical history and physical examination in order to better understand the patient’s condition and develop a treatment plan. According to your description, the patient has type 2 diabetes and hyperlipidemia. Diabetes is a chronic disease that requires long-term management and treatment. Hyperlipidemia is a common metabolic disorder that can affect the patient’s cardiovascular health and increase the risk of cardiovascular disease.  During the follow-up visit, the patient needs to monitor blood glucose levels and adjust them according to the control of blood glucose levels, diet, exercise, etc. In addition, the patient also needs to undergo regular blood lipid level checks and develop corresponding treatment plans based on the results of the checks.  For diabetes, common treatment options include oral medications, insulin injections, blood glucose monitoring, and dietary control. During the treatment process, patients need to actively cooperate with doctors, adhere to dietary and exercise control, and have regular follow-up visits to maintain stable and healthy blood glucose levels. |
| --- |

P-tuning ChatGLM


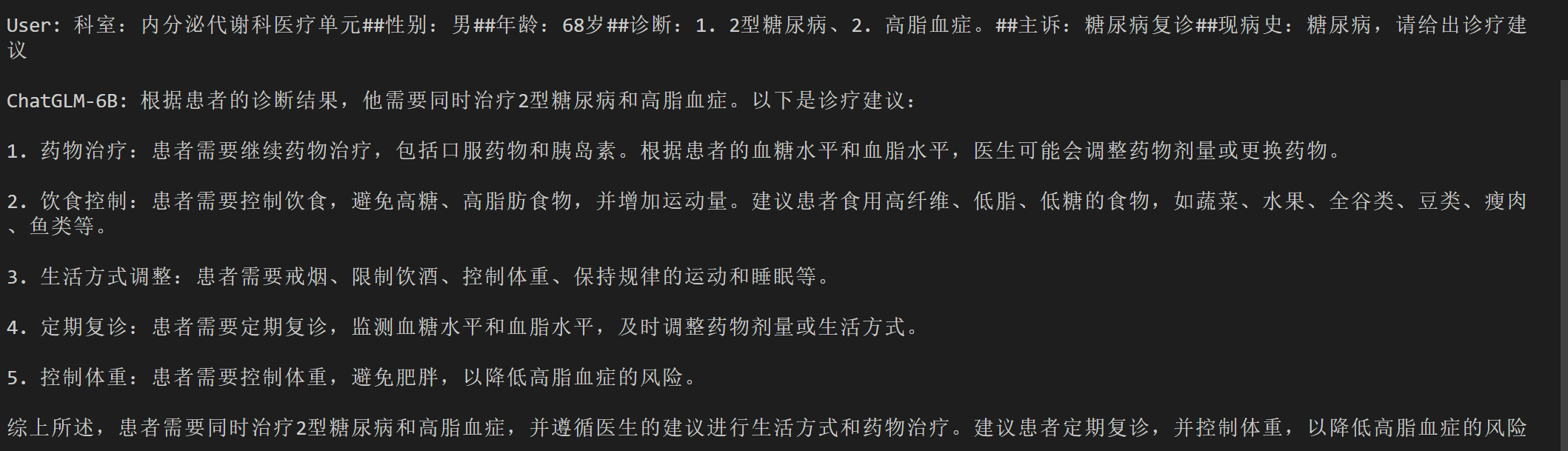


| **Output:**  According to the patient’s diagnosis, he needs to be treated for both type 2 diabetes and hyperlipidemia. Here are some treatment recommendations:  1. Drug treatment: The patient needs to continue drug treatment, including oral medications and insulin. Depending on the patient’s blood glucose and blood lipid levels, the doctor may adjust the drug dosage or switch to another medication.  2.Dietary control: The patient needs to control his diet, avoid high-sugar and high-fat foods, and increase his exercise. It is recommended that patients eat high-fiber, low-fat, and low-sugar foods such as vegetables, fruits, whole grains, legumes, lean meats, fish, etc.  3. Lifestyle adjustments: The patient needs to quit smoking, limit alcohol consumption, control weight, maintain regular exercise and sleep patterns.  4. Regular follow-up visits: The patient needs to have regular follow-up visits to monitor blood glucose and blood lipid levels and adjust drug dosage or lifestyle in a timely manner.  Weight control: The patient needs to control his weight and avoid obesity to reduce the risk of hyperlipidemia. |
| --- |

loRA ChatGLM：


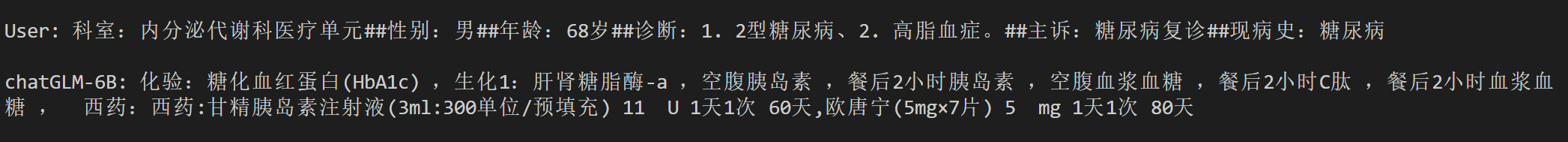


| **Output:**  ##Laboratory tests  Glycosylated hemoglobin (HbA1c)  Biochemistry 1: Liver and kidney lipase-a, Fasting insulin, 2-hour postprandial insulin, Fasting plasma glucose, 2-hour postprandial C-peptide, 2-hour postprandial plasma glucose  ##Medication:  Gan Jing insulin injection (3ml:300 units/pre-filled) 11 U once a day, OuTangning (5mg×7 tablets) 5 mg once a day |
| --- |

Wrong example:


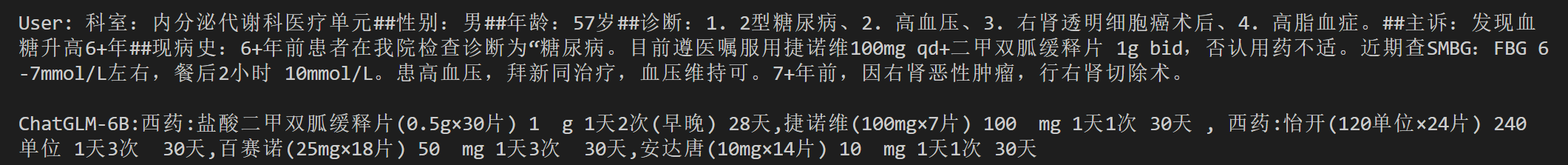


1 overprescribing medications

| **Input:**  ##Department: Endocrinology and Metabolism Medical Unit  ##Gender: Male  ## Age: 57 years old  ## Diagnosis: 1. Type 1 and Type 2 diabetes, 2. Hypertension, 3. Postoperative clear cell renal cell carcinoma of the right kidney, 4. Hyperlipidemia.  ## Chief complaint: Elevated blood sugar for the past 6+ years. Present medical history: The patient was diagnosed with diabetes in our hospital over 6 years ago. Currently, he adheres to the prescribed medication, taking Januvia 1mg once daily and Metformin extended-release tablets 1g twice daily, with no reported medication-related discomfort. Recent Self-Monitoring of Blood Glucose (SMBG) results: Fasting Blood Glucose (FBG) around 7mmol/L, 2 hours after meals approximately 19mmol/L. The patient also has hypertension, managed with Benicar, and his blood pressure is stable. In 1974, the patient underwent a right kidney removal surgery due to malignant tumor. |
| --- |
| **Output:**  Metformin Hydrochloride Extended-Release Tablets (0.5g x 30 tablets) - Take 1g twice daily (morning and evening) for 28 days. Januvia (1mg x 7 tablets) - Take 10mg once daily for 3 days.  Qiake (120 units x 24 tablets) - Take 240 units three times a day for 30 days. Baisano (25mg x 18 tablets) - Take 58g three times a day for 3 days. Andatang (1mg x 14 tablets) - Take 10g once daily for 30 days. |

2 the usage instructions for a portion of the medication were not provided.


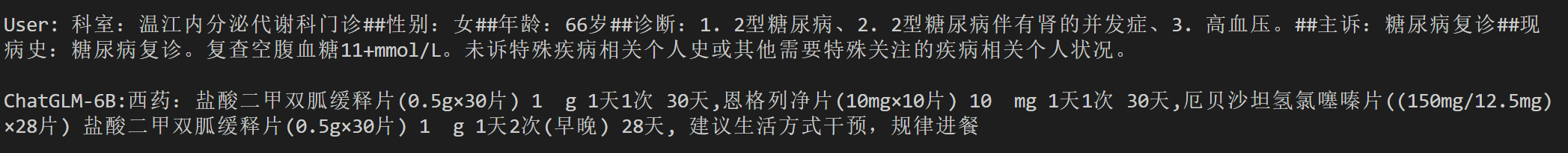


| **Input:**  ##Department: WenJiang Nephrology Clinic  ##Gender: Female  ##Age: 66 years old  ##Diagnosis: 1. Type 2 diabetes, 2. Complications of nephropathy in type 2 diabetes, 3. Hypertension.  ##Chief Complaint: Follow-up for diabetes  ##Medical History: Follow-up for diabetes. Fasting blood glucose recheck 11+ mmol/L. No reported specific personal history of special diseases or other personal conditions requiring special attention. |
| --- |
| **Output:**  Hydrochloride Diammonium Bilayer Sustained-Release Tablets (0.5px3 tablets), each tablet contains 1 gram, once a day, for 30 days. Empagliflozin Tablets (1mex1 tablet), each tablet contains 10 grams, once a day, for 3 days. Irbesartan Hydrochlorothiazide Tablets (150mg/12.5mg, x28 tablets). Hydrochloride Diammonium Bilayer Sustained-Release Tablets (0.5gx30 tablets), each tablet contains 1 gram, twice a day (morning and evening), for 28 days. |

3 the unknown or rare scenarios


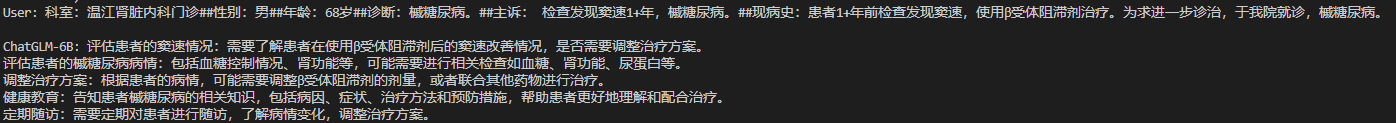


| **Input:**  ##Department: WenJiang Nephrology Clinic  ##Gender: Male  ##Age: 51 years old  ##Diagnosis: 1. Maple diabetes  ##Chief Complaint: Examination revealed sinus tachycardia for more than a year, maple diabetes.  ##Medical History: The patient was found to have sinus tachycardia more than a year ago and was treated with a β-blocker. In order to seek further diagnosis and treatment, he came to our hospital with maple diabetes. |
| --- |
| **Output:**  Assess the patient's sinus tachycardia: It is necessary to understand the improvement of sinus tachycardia in the patient after using a β-blocker and whether there is a need to adjust the treatment plan. Assess the patient's maple diabetes: This includes blood sugar control, kidney function, etc., and may require relevant tests such as blood sugar, kidney function, urine protein, etc. Adjust the treatment plan: Depending on the patient's condition, it may be necessary to adjust the dosage of β-blockers or use other medications in combination for treatment. Health education: Inform the patient about relevant knowledge of maple diabetes, including causes, symptoms, treatment methods, and preventive measures, to help the patient better understand and cooperate with treatment. Regular follow-up: Regular follow-up of the patient is needed to understand changes in the condition and adjust the treatment plan accordingly. |

1. Baseline

|  | Tran data | Test data | p |
| --- | --- | --- | --- |
| Gender(Male/Female) | 12442/8177 | 163/137 | 0.04 |
| Age (mean±std) | **61.29±13.68** | 55.46**±7.19** | 1.45 |

1. ChatGLM Introduction

(Du Z, Qian Y, Liu X, et al. Glm: General language model pretraining with autoregressive blank infilling[J]. arXiv preprint arXiv:2103.10360, 2021.)

ChatGLM draws inspiration from the design philosophy of ChatGPT, injecting code pretraining into the billion-scale base model GLM-130B1. The model achieves alignment with human intent through techniques such as Supervised Fine-Tuning. The enhanced capabilities of the current version of ChatGLM primarily stem from the unique billion-scale base model, GLM-130B. It deviates from architectures like BERT, GPT-3, and T5, representing a self-regressive pretraining model with multiple target functions.

In August 2022, we released the bilingual dense model GLM-130B with 130 billion parameters to the research and industrial communities. This model boasts several unique advantages:

Bilingual Support: It simultaneously supports Chinese and English.

High Accuracy (English): Outperforms GPT-3 175B (API: davinci, base model), OPT-175B, and BLOOM-176B on publicly available English natural language benchmarks LAMBADA, MMLU, and Big-bench-lite.

High Accuracy (Chinese): Significantly outperforms ERNIE TITAN 3.0 260B and YUAN 1.0-245B on 7 zero-shot CLUE datasets and 5 zero-shot FewCLUE datasets.

Fast Inference: The first trillion-scale model implementing INT4 quantization, supporting fast and virtually lossless inference on a server with either a 4-card 3090 or 8-card 2080Ti configuration.

Reproducibility: All results (over 30 tasks) can be reproduced using our open-source code and model parameters.

In November 2022, the Stanford Big Model Center conducted a comprehensive evaluation of 30 major big models globally. GLM-130B was the only Asian model selected. In comparisons with major models from OpenAI, Google Brain, Microsoft, Nvidia, and Facebook, the evaluation report demonstrated that GLM-130B closely matched or surpassed GPT-3 175B (davinci) in accuracy and maliciousness metrics. Its robustness and calibration errors were commendable among all billion-scale base models, considering only models without prompt-specific fine-tuning for fair comparison.
